# Incidence, Risk Factors, and Outcomes of De Novo Malignancy following Kidney Transplantation

**DOI:** 10.3390/jcm13071872

**Published:** 2024-03-24

**Authors:** Chukwuma A. Chukwu, Henry H.L. Wu, Kairi Pullerits, Shona Garland, Rachel Middleton, Rajkumar Chinnadurai, Philip A. Kalra

**Affiliations:** 1 Department of Renal Medicine, Northern Care Alliance NHS Foundation Trust, Salford M6 8HD, UK; chukwuma.chukwu@nhs.net (C.A.C.); rachel.middleton@nca.nhs.uk (R.M.); philip.kalra@nca.nhs.uk (P.A.K.); 2 Renal Research Laboratory, Kolling Institute of Medical Research, Royal North Shore Hospital, The University of Sydney, Sydney, NSW 2065, Australia; hon.wu@sydney.edu.au; 3 Faculty of Biology, Medicine & Health, The University of Manchester, Manchester M1 7HR, UK; kairi.pullerits@student.manchester.ac.uk (K.P.); shona.garland3@nhs.scot (S.G.)

**Keywords:** de novo post-transplant malignancy, kidney transplantation, immunosuppression, risk factors, outcomes

## Abstract

**Introduction:** Post-transplant malignancy is a significant cause of morbidity and mortality following kidney transplantation often emerging after medium- to long-term follow-up. To understand the risk factors for the development of de novo post-transplant malignancy (DPTM), this study aimed to assess the incidence, risk factors, and outcomes of DPTM at a single nephrology centre over two decades. **Methods:** This retrospective cohort study included 963 kidney transplant recipients who underwent kidney transplantation between January 2000 and December 2020 and followed up over a median follow-up of 7.1 years (IQR 3.9–11.4). Cox regression models were used to identify the significant risk factors of DPTM development, the association of DPTM with graft survival, and mortality with a functioning graft. **Results:** In total, 8.1% of transplant recipients developed DPTM, and the DPTM incidence rate was 14.7 per 100 patient-years. There was a higher mean age observed in the DPTM group (53 vs. 47 years, *p* < 0.001). The most affected organ systems were genitourinary (32.1%), gastrointestinal (24.4%), and lymphoproliferative (20.5%). Multivariate Cox analysis identified older age at transplant (aHR 9.51, 95%CI: 2.60–34.87, *p* < 0.001) and pre-existing glomerulonephritis (aHR 3.27, 95%CI: 1.10–9.77, *p* = 0.03) as significant risk factors for DPTM. Older age was significantly associated with poorer graft survival (aHR 8.71, 95%CI: 3.77–20.20, *p* < 0.001). When age was excluded from the multivariate Cox model, DPTM emerged as a significant risk factor for poor survival (aHR 1.76, 95%CI: 1.17–2.63, *p* = 0.006). **Conclusion:** These findings underscore the need for tailored screening, prevention, and management strategies to address DPTM in an aging and immunosuppressed kidney transplant population.

## 1. Introduction

Kidney transplantation is the optimal treatment for end-stage kidney disease (ESKD) and is recommended for all individuals who have no contraindications [1]. Immunosuppressive treatment is a necessary requirement for the longevity of transplant function. However, this comes at a cost of increased risk of infections and malignancies [2].

Ongoing research efforts to improve post-transplant infection and mitigation of cardiovascular risks are encouraged [3]. De novo post-transplant malignancy (DPTM) remains the most challenging of complications to diagnose and manage associated with immunosuppression and is the second-leading cause of mortality in the kidney transplant population following cardiovascular events [4]. The cumulative incidence of solid organ malignancies at 15 years following kidney transplantation is reported to be between 10 and 15% [5,6,7].

Given that infection with oncogenic viruses contributes to an increased risk of DPTM in transplant recipients, preventive policies, such as human papillomavirus (HPV) vaccination programs, have been introduced in some countries [8]. Routine cancer screening programs post-transplant have also been suggested to enable the earlier detection of DPTM [9]. Whilst these measures are now increasingly established within the clinical setting, a holistic understanding of the risk factors, time to diagnosis, and effects of DPTM on overall clinical outcomes remain incomplete. In order to provide further answers to these knowledge gaps, we conducted an observational study with more than 20 years of patient follow-up. Therefore, this study aimed to determine the predictors of time to DPTM, excluding non-melanoma skin cancers, and to evaluate the impact of DPTM on graft and patient survival post-transplantation.

## 2. Materials and Methods

### 2.1. Study Population

This was a retrospective cohort study of kidney allograft recipients who underwent kidney transplantation at a single tertiary nephrology center, namely the Northern Care Alliance NHS Foundation Trust, between 1 January 2000 and 31 December 2020. Eligible study participants were followed up until graft loss, death, loss to follow-up, or 31 December 2020. Subjects were excluded from the study if they lost their allograft within 3 months of kidney transplantation. Patients were classed as lost to follow-up when they had a change of address away from the catchment area covered by the Northern Care Alliance NHS Foundation Trust. The last clinic follow-up date was used as the endpoint for this group of patients. Patients with early graft loss (<6 months) and missing data were excluded from the analysis.

### 2.2. Data Collection

Data regarding demographic characteristics, pre-transplant comorbidities, transplant-related factors, virology status, biochemical parameters, and outcomes were extracted from electronic medical records including primary care and secondary care patient records. Variables of interest included age, gender, pre-transplant body mass index (BMI), ethnicity, smoking history, primary aetiology of kidney disease, pre-transplant comorbidities (e.g., diabetes mellitus, cardiovascular disease), duration of pre-transplant kidney replacement therapy, details of the transplant procedure (e.g., donor type, HLA mismatch, immunosuppressive regimen), virology status (e.g., Cytomegalovirus (CMV), Epstein Barr virus (EBV), Polyoma virus), biochemical parameters (e.g., tacrolimus level, baseline estimated glomerular filtration rate (eGFR), C-reactive protein (CRP), hemoglobin), and post-transplantation complications, such as any history of biopsy-proven acute graft rejection and any event of post-transplant viral infections and outcomes (e.g., DPTM incidence, death-censored graft loss, death with functioning graft). The underlying kidney disease that resulted in a referral for kidney transplantation was classified into autosomal dominant polycystic kidney disease; glomerulonephritis (GN); hypertensive kidney disease; diabetic kidney disease; reflux/chronic pyelonephritis; unknown aetiology; and ‘other’ etiologies of primary kidney disease (Supplementary Table S1). Routine pre-transplant cancer screening was performed as per the national transplant work-up guidelines.

Non-melanoma skin cancers, such as basal cell carcinoma and squamous cell carcinoma, were excluded from analysis because these cancers are typically less aggressive and have a significantly lower risk of metastasis compared to other types of malignancies. Excluding them from analysis enabled a focus on rarer and potentially more clinically significant malignancies that may have a greater impact on the prognosis of kidney transplant recipients. In patients with multiple cancer diagnoses, the date of the first cancer diagnosis was taken as the date of DPTM. Recipients’ laboratory data were obtained from the final laboratory tests recorded before the end of each year of follow-up and were recorded until the conclusion of the observation period. The average of these observations was determined by calculating the median value of each laboratory parameter.

### 2.3. Local Post-Transplant Immunosuppression Protocol

The immunosuppression protocol at the regional transplant centre includes induction with an interleukin-2 receptor antagonist (i.e., Basiliximab) for all patients except those who were recipients of multi-organ transplantation (which consisted of only 2 patients who received simultaneous pancreas and kidney transplantation). These patients received anti-CD52 induction therapy (i.e., Alemtuzumab) instead. A maintenance immunosuppressive regimen consisted of calcineurin inhibitor-based immunosuppression (i.e., either Tacrolimus or Cyclosporine). Individuals without contraindications also received an anti-proliferative agent (i.e., usually Mycophenolic acid and, less frequently, Azathioprine).

Regarding corticosteroid maintenance, kidney transplant recipients with a standard immunologic risk profile received <2 weeks of corticosteroid treatment. Those considered to have a higher immunologic risk profile, such as younger transplant recipients and/or older organ donors, calculated panel reactive antibody >20%, the presence of a donor-specific antibody, delayed graft function, HLA incompatible transplant, and prolonged ischemia time >24 h, received a longer corticosteroid treatment course. Recipients receiving corticosteroids were reviewed after 3 and 6 months to decide whether corticosteroids could be discontinued.

### 2.4. Statistical Analysis

Descriptive statistics including means, standard deviations, medians, interquartile ranges, counts, and percentages were used to summarize the study data. Continuous variables were expressed as mean and standard deviation (SD) or median and interquartile range (IQR), depending on the distribution’s normality. Categorical variables were presented as frequency (in %). Statistical differences between data groups were assessed using Chi-squared or Fisher’s exact test for categorical variables and Student’s *t*-test or Wilcoxon sign-rank test for parametric and non-parametric numerical variables, respectively.

The determination of the slope of eGFR referred to as delta eGFR (ΔeGFR) involved utilizing a linear mixed-effects regression model. The model was constructed using annually obtained eGFR results specific to each transplant recipient. The final eGFR value recorded at the end of each follow-up year was utilized in the statistical model until the end of the entire observation period. To ensure the model’s robustness, a minimum of 3 eGFR values were considered to calculate an individual’s ΔeGFR. The final eGFR recorded for each year’s follow-up was chosen because it would be a representative endpoint encapsulating the overall kidney function at the conclusion of each year, ensuring uniformity in the analysis across study participants.

The outcomes of interest included the incidence and risk predictors of time to DPTM as well as the impact of DPTM on allograft and recipient survival. Univariate and multivariate Cox proportional hazard regression analyses were performed to identify significant risk factors associated with the time to DPTM development. Hazard ratios (HRs) with 95% confidence intervals (CIs) were reported. Predictor variables included in the univariate model included patient age (years) at the time of kidney transplantation; sex; ethnicity; primary aetiology of underlying kidney disease; pre-transplant cytomegalovirus status (for both donor and recipient) and organ donor status; event of pre-emptive transplantation; post-transplant immunosuppression agent classes prescribed; baseline eGFR; history of biopsy-proven acute graft rejection; smoking history post-transplantation; and post-transplant DNA viral infection status. Variables demonstrating a correlation at a significance level of *p* < 0.20 in the univariate analysis were incorporated into the multivariate model for time from kidney transplantation to DPTM.

Evaluating the impact of post-transplant malignancy on both graft and recipient survival, we employed a comprehensive approach utilizing Kaplan–Meier analysis, Cox regression, and competing risk regression. The selection of confounding variables for incorporation into the multivariate Cox regression and competing risk regression models for determinants of graft and recipient survival was guided by existing knowledge regarding the potential risk factors that influence these post-transplant clinical outcomes. To assess the effect of DPTM on death-censored graft loss, the confounding variables included primary aetiology of the native kidney disease, gender, history of acute rejection, donor type, total HLA mismatch, and baseline eGFR. Due to the relationship between age and DPTM, an age-adjusted model and an age-unadjusted model were created. Similarly, to evaluate the effect of DPTM on recipient death, the adjusted confounders include recipient age, gender, primary aetiology of kidney disease, pre-emptive transplantation, and baseline eGFR. Again, due to the collinear relationship between recipient age and DPTM, two Cox regression models were created: an age-adjusted model and an age-unadjusted model.

Additionally, to account for the presence of competing risks inherent within the transplantation setting, competing risk regression models of the Fine and Grey methods were employed [10]. These models were used to consider the occurrence of multiple possible events, such as graft failure or death, as competing risks in the presence of post-transplant malignancy. Adjustments were made for the same confounding variables used in the Cox regression model.

Statistical significance was set at *p* < 0.05. All statistical analyses were conducted using R software (R Foundation for Statistical Computing, Vienna, Austria, version 4.2.2). The total percentage of missing data was 5.74%. In this study, missing data were handled using listwise deletion.

### 2.5. Ethical Considerations of the Study

This study was conducted following the principles outlined in the Declaration of Helsinki. Ethical approval was obtained from the institutional review board of the Northern Care Alliance NHS Foundation Trust (reference number: S21HIP03). Patient consent was not required as this study was based on publicly available data. The need for informed consent was waived by the Greater Manchester South Research Ethics Committee in the United Kingdom.

## 3. Results

**Figure 1:**
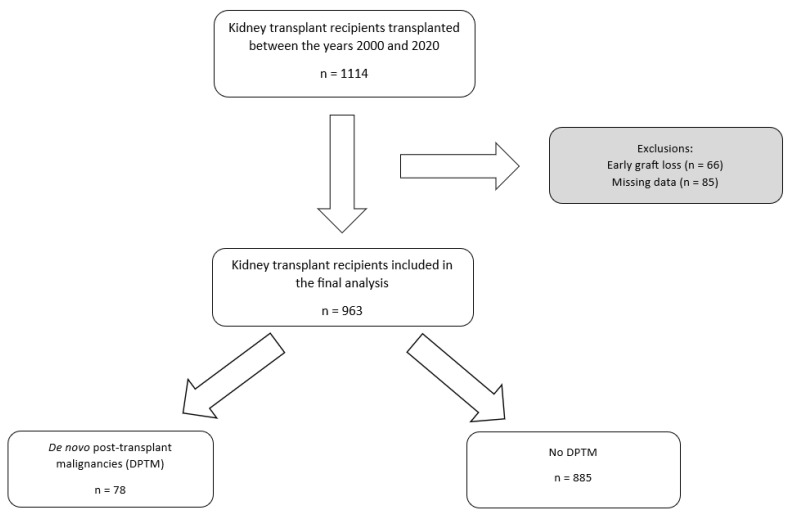
Flowchart of the patient cohort selection process. DPTM: De novo post-transplant malignancy; KTR: Kidney transplant recipients.

A total of 1114 kidney transplant recipients between 1 January 2000 and 31 December 2020 were examined for eligibility in this study. After excluding 151 recipients due to early graft loss (66 patients) and missing data (85 patients), 963 recipients were included in the final analysis. The total follow-up period amounted to 7475 patient-years. The median follow-up time was 7.1 years (IQR 3.9–11.4). In total, 78 (8.1%) patients had DPTM during this follow-up period. The DPTM incidence rate was 14.7 per 100 patient-years. The flowchart of patient recruitment in this study is illustrated (Figure 1).

The mean age of the cohort was 47 years, with age being significantly higher in transplant recipients with DPTM (53 vs. 47 years, *p* < 0.001). A longer duration of steroid maintenance (i.e., >6 months) (60 vs. 46%, *p* = 0.027) and presence of EBV infection (29.5 vs. 13%, *p* < 0.001) were reported with greater frequency amongst transplant recipients with DPTM. The link between EBV infection and malignancy predominantly stems from a correlation with lymphoproliferative malignancies. Among transplant recipients who had EBV infection, the incidence of lymphoproliferative malignancy was 14 times higher than their counterparts who remained uninfected (9.8% vs. 0.7%). Transplant recipients with other solid organ malignancies also had increased EBV infection but with a comparatively lower ratio of 1.8 times compared to recipients without EBV infection (11.4% vs. 6.3%). Transplant recipients who developed DPTM had significantly lower mean hemoglobin (121 vs. 126 g/L, *p* = 0.015) and a higher baseline median C-reactive protein (31 vs. 14 mg/L, *p* < 0.001). The mortality rate with a functioning graft was higher in transplant recipients who developed DPTM (42.3 vs. 19%, *p* < 0.001) (Table 1).

The distribution of organs and organ systems involved in DPTM is shown in Supplementary Table S2. Genitourinary (32.1%), gastrointestinal tract (24.4%) and lymphoproliferative (20.5%) systems were the most common organ systems affected by DPTM. The median time for the occurrence of DPTM was 6.5 years (IQR 3.1–10.2) following kidney transplantation (Supplementary Figure S1).

**Figure 2:**
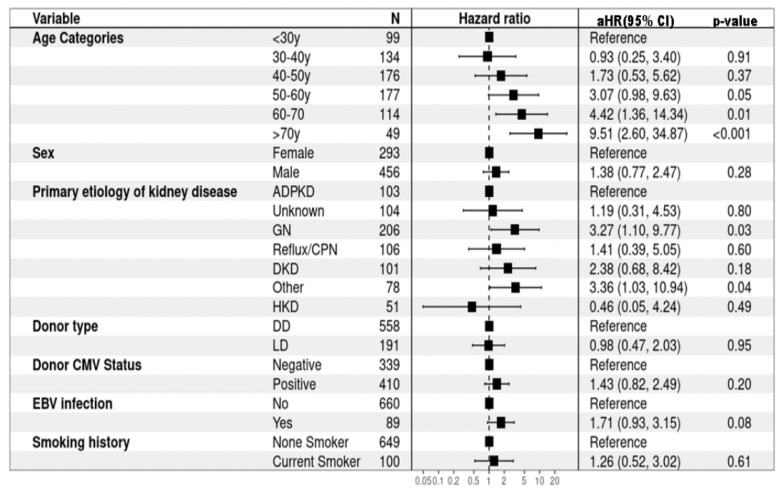
Risk factors for de novo post-transplant malignancy (multivariate Cox proportional hazard regression). ADPKD: Autosomal dominant polycystic kidney disease; CMV: Cytomegalovirus; CPN: Chronic pyelonephritis; DD: Deceased donor; DKD: Diabetic kidney disease; EBV: Epstein–Barr virus; GN: Glomerulonephritis; HKD: Hereditary kidney disease; LD: Living donor.

In the univariate Cox regression analysis, several factors were identified as significant risk factors for the development of DPTM, including older age at the time of kidney transplant (HR 1.59, 95%CI: 1.34–1.88, *p* < 0.001), higher median C-reactive protein (CRP) levels (HR 1.02, 95%CI: 1.01–1.02, *p* < 0.001), and EBV infection (HR 2.09, 95%CI: 1.28–3.41, *p* = 0.003) (Supplementary Table S3). In the multivariate model, age >60 years at the time of kidney transplantation emerged as a significant risk factor for the development of DPTM. Specifically, individuals aged 60–70 years demonstrated an aHR of 4.42 (95%CI: 1.36–14.34, *p* < 0.001), while those aged over 70 years exhibited a substantially higher risk, with an aHR of 9.51 (95%CI: 2.60–34.87, *p* < 0.001). Additionally, primary etiologies of kidney disease were identified as significant risk factors, with pre-existing GN (aHR 3.27, 95%CI: 1.10–9.77, *p* = 0.03) and primary etiologies classified in the ‘other’ category (aHR 3.36, 95%CI: 1.03–10.94, *p* = 0.04) showing statistically significant associations with DPTM development (Figure 2). However, caution is warranted when interpreting study findings relating to etiologies listed in the ‘other’ category of primary kidney diseases, as this comprises a heterogeneous group of pathologies (Supplementary Table S1), which potentially introduces variability and complexity into the analysis, making it difficult to draw definitive conclusions from results displayed here.

**Figure 3:**
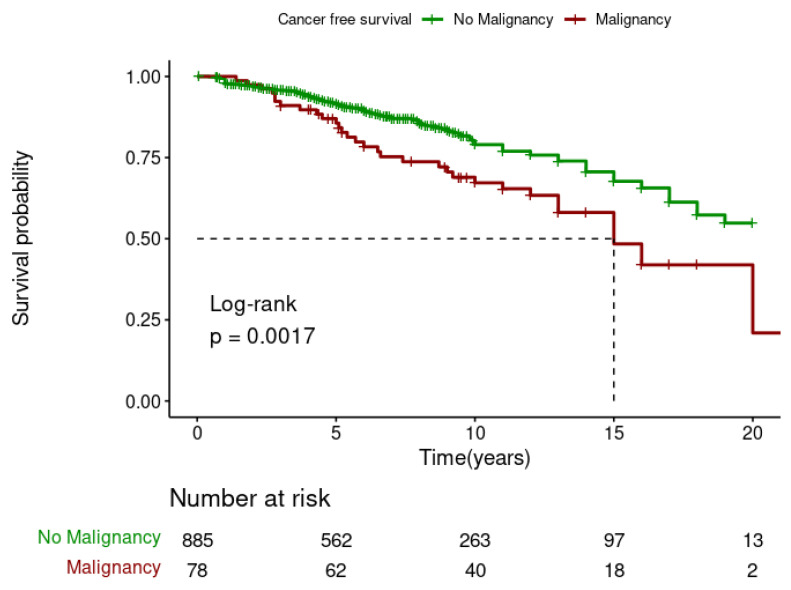
Effect of de novo post-transplant malignancy on recipient survival.

**Figure 4:**
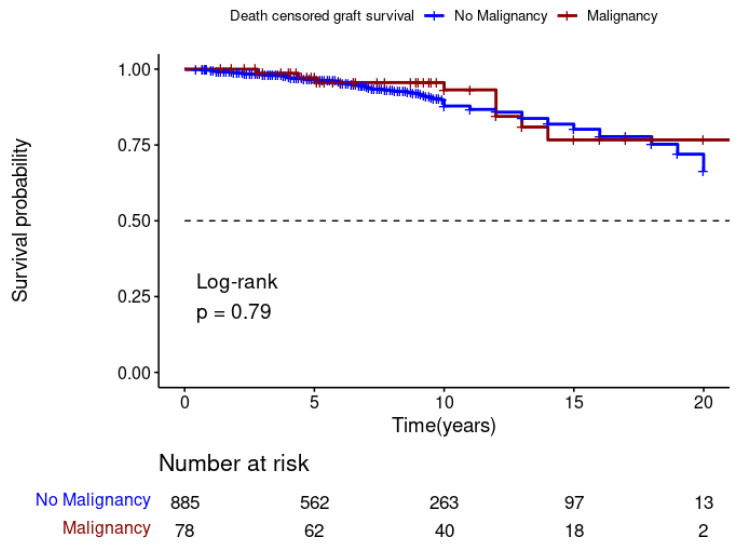
Effect of de novo post-transplant malignancy on death-censored graft survival.

The Kaplan–Meier analysis showed that DPTM was associated with poor survival for kidney transplant recipients (Log-rank; *p* = 0.001) (Figure 3), but it was not associated with death-censored graft survival (Log-rank; *p* = 0.79) (Figure 4). The 5-, 10-, and 15-year transplant recipient survival was 92%, 79% and 67%, respectively, in the non-cancer group compared to 86%, 68%, and 48% amongst those who developed DPTM.

**Figure 5:**
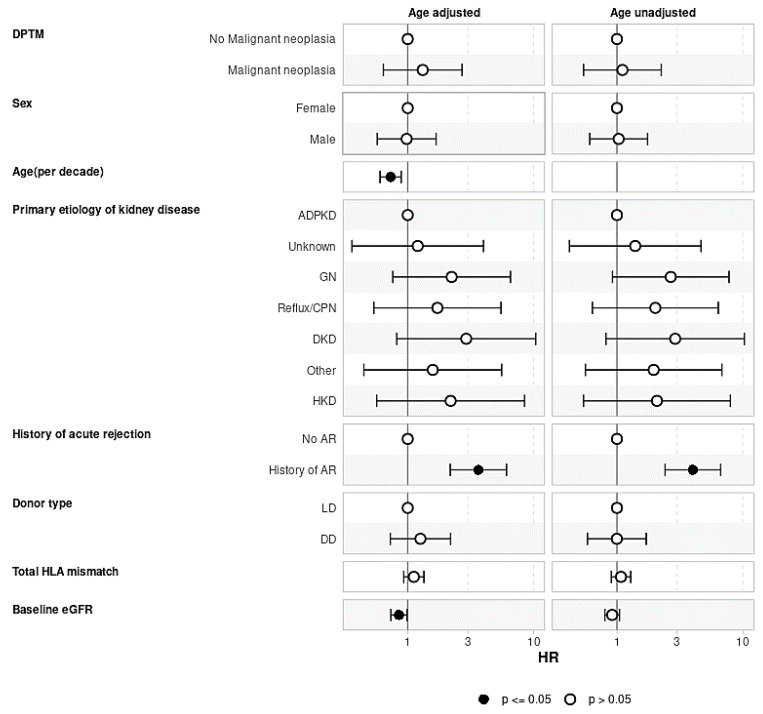
Multivariate Cox regression analysis evaluating the association of de novo post-transplant malignancy with graft loss. The effect of DPTM on death-censored graft loss adjusted for factors known to be associated with graft loss, including native kidney disease, recipient age, gender, history of acute rejection, donor type, total HLA mismatch, and baseline eGFR. There was no difference in graft loss censored for death in recipients who developed DPTM vs. those who did not develop DPTM. ADPKD: Autosomal dominant polycystic kidney disease; AR: Acute rejection; CPN: Chronic pyelonephritis; DD: Deceased donor; DKD: Diabetic kidney disease; DPTM: De novo post-transplant malignancy; eGFR: Estimated glomerular filtration rate; GN: Glomerulonephritis; HR: Hazard ratio; HKD: Hereditary kidney disease; LD: Living donor.

**Figure 6:**
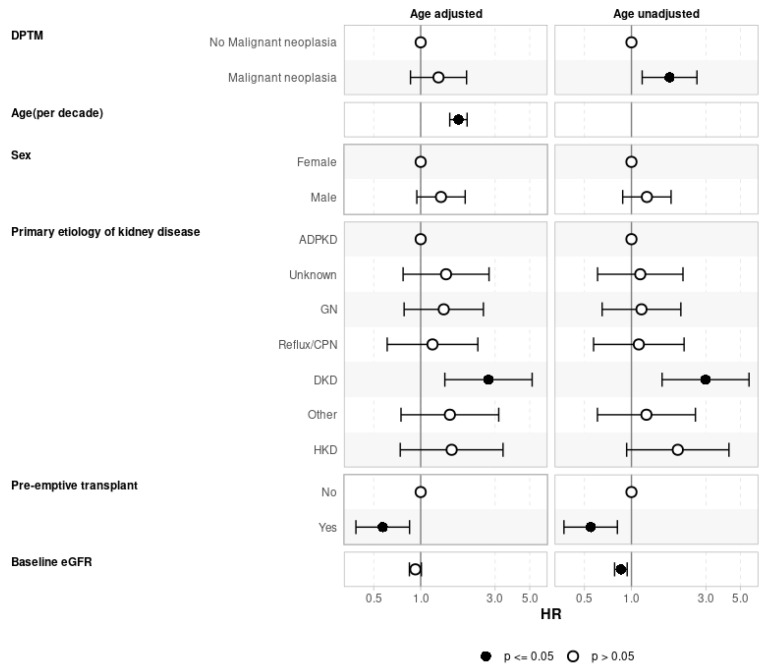
Association of de novo post-transplant malignancy on recipient survival adjusted for confounders (multivariate Cox regression analysis). The effect of DPTM on recipient survival adjusted for recipient age, gender, native kidney disease, pre-emptive transplantation, and baseline eGFR. The age-adjusted model did not show any difference in recipient survival between the DPTM group and the no DPTM group whereas the age-unadjusted model showed an increased hazard of death with DPTM. ADPKD: Autosomal dominant polycystic kidney disease; CPN: Chronic pyelonephritis; DKD: Diabetic kidney disease; DPTM: De novo post-transplant malignancy; eGFR: Estimated glomerular filtration rate; GN: Glomerulonephritis; HKD: Hereditary kidney disease.

Evaluating the effect of DPTM on graft loss (Figure 5) and recipient death (Figure 6), the Cox regression models were adjusted for confounding variables, as shown in the figures. Both the age-adjusted and age-unadjusted Cox model did not show any difference in death-censored graft loss between the transplant recipients who developed DPTM and those who did not (age-adjusted model: aHR 1.32, 95%CI: 0.64–2.70, *p* = 0.455; age-unadjusted model: aHR 1.10, 95%CI: 0.54–2.25, *p* = 0.783). In regard to transplant recipient death outcomes, the age-adjusted multivariate Cox regression model did not demonstrate any difference in transplant recipients’ death between transplant recipients with DPTM and those who did not (aHR 1.30, 95%CI: 0.86–1.97, *p* = 0.212). However, in the age-unadjusted multivariate Cox regression model, death amongst transplant recipients with DPTM was observed to be significantly higher compared to those who did not (aHR 1.76, 95%CI: 1.17–2.63, *p* = 0.006).

In the competing risk analysis, the cumulative incidence of death-censored graft loss and recipient death stratified by the presence or absence of DPTM at specific timepoints at 5-year, 10-year, and 15-year follow-up were estimated (Supplementary Tables S4 and S5). Among patients who developed DPTM, the cumulative incidence of death-censored graft loss at 5-year, 10-year, and 15-year follow-up timepoints was 2.6%, 5.7%, and 16%, respectively. This was not significantly different (*p* = 0.50) from the cumulative incidence of death-censored graft loss amongst those who did not develop DPTM, which was 3.4%, 11% and 17%, respectively, at 5-year, 10-year, and 15-year follow-up timepoints. On the other hand, the cumulative incidence of transplant recipients’ death was significantly higher amongst those who developed DPTM, which was 14%, 32%, and 48% at 5-year, 10-year, and 15-year follow-up compared to those who did not develop DPTM, which was 8.3%, 20%, and 30% at 5-year, 10-year, and 15-year follow-up (*p* = 0.002).

In the competing risk regression model of graft loss with transplant recipient death as the competing risk (Supplementary Table S6), the results were similar to that of the Cox regression analysis. After adjusting for recipient age, gender, primary aetiology of kidney disease, donor type, history of acute rejection, total HLA mismatch, and baseline eGFR, there was no significant difference in graft loss between those who developed DPTM and those who did not (age-adjusted model: sub-hazard ratio (SHR) 1.31, 95%CI: 0.70–2.49, *p* = 0.40; age-unadjusted model: SHR 1.03, 95% CI: 0.53–2.02, *p* = 0.920). In the analysis of transplant recipient death with graft loss as the competing risk (Supplementary Table S7), the results were similar to that observed in the Cox regression model. The age-unadjusted model demonstrated a significantly higher risk of transplant recipient death amongst those who developed DPTM (SHR 1.78, 95%CI: 1.19–2.66, *p* = 0.005), but the age-adjusted model did not illustrate significant differences in transplant recipient death between the two groups.

## 4. Discussion

The observed incidence rate of DPTM in our cohort, 14.7 per 100 patient-years, representing 8% of patients during a median follow-up period of 7.1 years, was very similar to the range reported in the current literature, which typically falls between 10% and 15% [4,5,6,7]. Any disparity in incidence rates in the literature may be attributed to differences in the distribution of organs and organ systems affected by DPTM, as well as geographical variations in cancer epidemiology among kidney transplant recipients. It is reported that viral-related and immune-driven cancers, such as post-transplant lymphoproliferative disorder (PTLD), anogenital cancers, and Kaposi’s sarcoma, are commonest following kidney transplantation, whilst the risk of certain cancers, such as prostate, breast, colorectal, lung, melanoma, thyroid, gynecological, and kidney cancers, either remained unchanged or exhibited only mild increases in kidney transplant recipients [11,12]. In our study, however, urogenital, gastrointestinal cancers, and PTLD were the most frequently detected DPTMs. This differential risk profile highlights the complex interplay between immunosuppression, viral infections, and organ-specific and recipient-specific susceptibilities.

Previous reports have revealed geographical variations in the distribution of organs and organ systems affected by DPTM, with higher incidences of de novo urothelial cancers, renal cell carcinomas, and gastrointestinal cancers reported in non-Western Asian and Middle Eastern cohorts compared to European and North American populations [5], possibly reflecting underlying genetic predispositions, environmental exposures, or regional differences in healthcare practices. Our cohort of patients contains a high proportion of recipients with Asian and Middle Eastern ethnicity.

Amongst the European, North American, Australian, and New Zealand White populations, the most common DPTMs are non-melanoma skin cancers, PTLD, and lip cancer [6,13,14,15]. Population studies have reported an elevated risk of liver cancer among kidney transplant recipients in the Taiwanese population, which has been attributed to the influence of endemic chronic hepatitis B virus (HBV) and hepatitis C virus (HCV) infections in certain ethnic populations [16]. Furthermore, differences in dietary patterns have been linked to higher incidence of certain types of cancers. For example, intake of aristolochic acid has been associated with increased post-transplant urothelial carcinoma [17].

The association between age and DPTM risk observed in our study was not surprising and aligns with the existing literature [2,18]. Additionally, a higher inflammatory state (i.e., higher median CRP) and EBV infection were noted as risk potential factors for DPTM in the univariate analysis but not in the multivariate analysis. Other reported risk factors by previous studies include long-term immunosuppressive use, occurrence of acute graft rejection, sensitization status, and duration of dialysis pre-transplantation [19,20,21].

There is emerging evidence that kidney function decline is an independent prognosticating factor for cancer development after kidney transplantation [22,23]. Increased DNA damage as a result of impaired DNA repair or reduced antioxidant capacity with kidney function decline have been implicated as the underlying mechanism of carcinogenesis [24]. Cancer risk in transplanted patients with kidney function decline is compounded if the individual had previous malignancy pre-transplantation [25,26]. Worsened clinical outcomes in DPTM would not be attributed to a previous history of malignancy alone [27,28].

Once DPTM occurs, our findings and those of previous studies highlight the heightened risk of mortality in affected individuals with cancer-associated mortality rates 5 to 10 times higher than the general population without a transplant [29]. The aetiology of this increased mortality risk is multifaceted, encompassing long-term immunosuppression, post-transplant infections, and suboptimal adherence to recommended prevention and screening strategies [30].

In relation to immunosuppression, the absence of conclusive evidence regarding the differential oncogenicity of various immunosuppressive agents highlights the need for further research to elucidate their respective roles in DPTM development and mortality outcomes [31]. Previous studies have historically shown that tacrolimus elevates levels of transforming growth factor-beta (TGF-β), thereby promoting cancer progression and metastatic disease in post-transplant hepatocellular carcinoma, human lung adenocarcinoma cells, and renal cell carcinoma [32]. In addition, calcineurin inhibitors also exhibit inhibitory signaling through calcineurin and nuclear factor of activated T-cells, resulting in p53 activation, a crucial checkpoint in the progression of non-melanoma skin cancer [33]. In addition, cyclosporine, through the TGF-β and IL-6 overexpression pathways, directly contributes to tumor development and progression by affecting immune system downregulation. It inhibits DNA repair, leading to mutations that induce apoptosis in activated T-cells, while inhibiting apoptotic processes in other cell types due to opened mitochondrial permeability transition pores [34,35]. Azathioprine sensitizes the skin to ultraviolet A radiation and leads to DNA accumulation of 6-thioguanine [36]. T-cell depleting agents, such as anti-thymocyte globulin and anti-CD52 monoclonal antibody, have historically been associated with an increased risk of DPTM, particularly PTLD and melanoma [37]. The development of cancer in this context is attributed to incomplete T-cell recovery following T-cell depletion, leading to disruptions in immune system homeostasis and subsequent cancer development [38,39,40]. Despite their limitations, mammalian target of rapamycin (mTOR) inhibitors may exhibit potential anti-tumor effects by inhibiting cancer growth through cell-cycle arrest and initiation of apoptosis [41].

Oncogenic virus-induced cancer development post-transplantation is primarily associated with compromised immune control. The suppression of immune function can lead to an accumulation of mutations that would normally be taken care of by an intact immune system [42].

The debate surrounding the benefits of implementing routine screening and prevention strategies for DPTM persists, prompting questions about the impact of uptake and adherence to these measures on DPTM development [43]. While numerous high-quality randomized clinical trials have demonstrated the efficacy of routine cancer screening in reducing cancer-specific mortality in the general population, the situation is different for patients with pre-existing kidney disease, such as GN. It is recognized that individuals with GN may experience accelerated development of cancerous changes and shorter survival times post-cancer diagnosis, potentially questioning the cost-effectiveness and substantial benefits of routine screening [44,45]. Although the independent risk factors for cancer development in GN patients are not yet fully understood, it is presumed to be primarily associated with long-term immunosuppression [46]. Despite the limited randomized data supporting routine screening in the post-transplant population, current guidelines recommend adhering to population-based cancer screening protocols for breast, colorectal, and cervical cancers, aligning with recommendations for the general population [47]. For kidney transplant recipients with cystic kidney disease, heavy smokers, and those using analgesics long-term, annual or biannual ultrasound of their native kidneys is advised to monitor for DPTM, particularly in relation to renal cell carcinoma [48]. Ultimately, decisions regarding cancer screening and post-transplant care should be personalized, considering each patient’s overall health, well-being, and available support. A collaborative decision-making process between patients and clinicians incorporating individual values and preferences is recommended when making informed decisions about the screening approach.

The observed multicollinear relationship between age, DPTM occurrence, and graft survival has not previously been highlighted. Previous studies have identified specific cancer types, treatment modalities, and response to treatment as significant predictors of graft survival following DPTM diagnosis [49,50] However, the optimal management strategies for balancing cancer control and graft preservation remain areas of ongoing investigation, thus necessitating personalized approaches tailored to individual patient characteristics and treatment responses.

The primary strength of our study lies in the comprehensive compilation of both pre-transplant and post-transplant demographic and clinical data spanning a 20-year period. This extensive dataset facilitated a detailed investigation into the long-term incidence and clinical implications of DPTM. Our analysis benefited from detailed information on time to diagnosis, the prescribed immunosuppressive regimen, occurrences of graft rejection, and various laboratory parameters, thus enabling a comprehensive exploration of risk factors and confounding variables.

However, several limitations of our study warrant acknowledgment. Firstly, its retrospective nature introduces the potential for incomplete records and study biases, although the extended period of patient follow-up partially mitigates this limitation. Additionally, being a single-center study may limit the generalizability of our findings to broader populations, given the variations in patient demographics, lifestyle habits, and healthcare practices across different regions. Thirdly, our data on the immunosuppression regimen were based on prescriptions at the time of hospital discharge following kidney transplantation without accounting for subsequent changes over time. Fourthly, whilst our analysis revealed a statistically significant association between the ‘other’ category of primary kidney disease and DPTM development, the lack of specificity regarding the underlying heterogeneous pathologies hinders our ability to elucidate the mechanisms driving this observed association. Therefore, this finding should be interpreted cautiously. Further investigation with larger sample sizes and detailed characterization of specific etiologies within the ‘other’ category are warranted. Additionally, subgroup analyses focusing on individual pathologies within this heterogeneous group may elucidate potential risk factors and mechanisms underlying DPTM development in these patients. Finally, whilst our analysis shows a clear difference in patient survival between the groups with and without DPTM, it is important to note that not all deaths were attributed to malignancy. Specific information on the cause of death for each patient was not consistently available or reliably documented in our dataset. This reflects challenges inherent to retrospective studies that are conducted over a long follow-up period, where data completeness and accuracy can vary. As such, the survival curves represent overall patient survival, regardless of the cause of death.

## 5. Conclusions

The incidence of DPTM in our cohort, at 14.7 per 100 patient-years, surpasses that of the general population. Advanced age and GN as the aetiology of ESKD emerged as independent predictors of DPTM. Crucially, our findings reveal that DPTM is associated with poorer survival outcomes among kidney transplant recipients, highlighting the need for risk-based screening and surveillance, particularly in older patients and those with a history of pre-transplant GN. Future research initiatives aimed at creating diverse cancer screening protocols for kidney transplant recipients are needed to enhance surveillance and facilitate early diagnosis of DPTM.

## Figures and Tables

**Table 1:** Baseline demographic and clinical characteristics of the study cohort.

Characteristics	No DPTM (n = 885)	DPTM (n = 78)	Total (n = 963)	*p*-Value
Age (years); mean (SD)	46.8 (15.3)	53.0 (13.6)	47.3 (15.2)	<0.001
Gender male	541 (61.1%)	56 (71.8%)	597 (62.0%)	0.063
Pre-transplant BMI; mean (SD)	27.1 (8.0)	26.8 (5.2)	27.1 (7.8)	0.785
Ethnicity				0.421
White	715 (80.8%)	69 (88.5%)	784 (81.4%)	
Black	18 (2.0%)	1 (1.3%)	19 (2.0%)	
Asian	129 (14.6%)	7 (9.0%)	136 (14.1%)	
Other	23 (2.6%)	1 (1.3%)	24 (2.5%)	
Smoking history				
Non-smoker	700 (85.9%)	64 (87.7%)	764 (86.0%)	0.674
Current smoker	115 (14.1%)	9 (12.3%)	124 (14.0%)	
Primary aetiology of kidney disease				0.103
ADPKD	120 (13.6%)	6 (7.7%)	126 (13.1%)	
Glomerulonephritis	238 (26.9%)	30 (38.5%)	268 (27.8%)	
Diabetic kidney disease	113 (12.8%)	8 (10.3%)	121 (12.6%)	
Hypertensive kidney disease	65 (7.3%)	3 (3.8%)	68 (7.1%)	
Reflux/chronic pyelonephritis	130 (14.7%)	7 (9.0%)	137 (14.2%)	
Unknown	130 (14.7%)	12 (15.4%)	142 (14.7%)	
Other	89 (10.1%)	12 (15.4%)	101 (10.5%)	
Pre-transplant co-morbidities				
Diabetes mellitus	153 (17.3%)	14 (17.9%)	167 (17.3%)	0.883
Cardiovascular disease	202 (22.8%)	12 (15.4%)	214 (22.2%)	0.130
Duration of pre-transplant KRT (months), mean (SD)	24.7 (33.6)	25.7(44.3)	24.8 (34.6)	0.445
Details relating to transplant procedure				
Transplant number; mean (SD)	1.15 (0.42)	1.12 (0.32)	1.14 (0.41)	0.520
Pre-emptive transplant	263 (31.2%)	21 (28.0%)	284 (30.9%)	0.566
Organ donor status				
DD	624 (70.7%)	60 (76.9%)	684 (71.2%)	0.242
LD	259 (29.3%)	18 (23.1%)	277 (28.8%)	
Total HLA mismatch; mean (SD)	2.4 (1.4)	2.4 (1.5)	2.4 (1.4)	0.763
Total ischaemia time; mean (SD)	12.8 (7.6)	13.0 (7.0)	12.8 (7.6)	0.770
History of acute rejection	100 (11.3%)	7 (9.0%)	107 (11.1%)	0.531
Post-transplant diabetes	147 (16.6%)	10 (12.8%)	157 (16.3%)	0.385
Primary immunosuppression				
Tacrolimus	788 (90.1%)	66 (88.0%)	854 (89.9%)	0.516
Cyclosporine	77 (8.8%)	7 (9.3%)	84 (8.8%)	
Sirolimus	10 (1.1%)	2 (2.7%)	12 (1.3%)	
Mycophenolic acid	683 (78.1%)	49 (64.5%)	732 (77.1%)	
Azathioprine	107 (12.2%)	16 (21.1%)	123 (12.9%)	
No anti-metabolite	84 (9.6%)	11 (14.5%)	95 (10.0%)	
Steroid maintenance				0.027
<2 weeks	466 (53.0%)	30 (38.5%)	496 (51.8%)	
2 weeks–6 months	22 (2.5%)	1 (1.3%)	23 (2.4%)	
>6 months	392 (44.5%)	47 (60.3%)	439 (45.8%)	
CMV status				
Donor (D) CMV negative	340 (46.4%)	21 (33.9%)	361 (45.5%)	0.056
Recipient (R) CMV negative	436 (59.0%)	33 (55.9%)	469 (58.8%)	0.645
Combined donor and recipient CMV status				0.097
CMV D + R-	127 (18.4%)	16 (29.1%)	143 (19.1%)	
CMV D-R-	162 (23.4%)	9 (16.4%)	171 (22.9%)	
CMV D + R+	241 (34.8%)	22 (40.0%)	263 (35.2%)	
CMV D-R+	162 (23.4%)	8 (14.5%)	170 (22.8%)	
Virology status				
CMV viremia	120 (13.6%)	13 (16.7%)	133 (13.8%)	0.446
EBV viremia	100 (11.3%)	23 (29.5%)	123 (12.8%)	<0.001
Polyoma viremia	111 (12.5%)	6 (7.7%)	117 (12.1%)	0.209
Any DNA virus infection	258 (29.2%)	30 (38.5%)	288 (29.9%)	0.085
Biochemical parameters				
Tacrolimus level (ng/mL); Mean (SD)	5.53 (2.74)	5.40 (3.34)	5.53 (2.79)	0.704
Baseline eGFR (mL/min/1.73 m^2^); median (IQR)	51.0(40.0–65.0)	47(37.7–58.0)	51.0 (40.0–64.0)	0.072
eGFR slope (mL/min/1.73 m^2^/year); mean (SD)	−1.35 (4.05)	−0.90 (2.44)	−1.31 (3.94)	0.371
Haemoglobin (g/L); mean (SD)	126.6 (19.5)	120.8 (17.1)	126.1 (19.4)	0.015
uPCR (mg/mmol); median (IQR)	23.8(11.6–70.8)	26.7(16.3–53.1)	24.2(11.8–68.4)	0.712
CRP (mg/dL); median (IQR)	12.6(4.0–32.6)	31.3(13.6–50.2)	14.0(4.1–34.4)	<0.001
Outcomes				
Death-censored graft loss	78 (8.8%)	10 (12.8%)	88 (9.1%)	0.239
Death with functioning graft	149 (16.8%)	33 (42.3%)	182 (18.9%)	<0.001

ADPKD: Autosomal dominant polycystic kidney disease; CMV: Cytomegalovirus; CRP: C-reactive protein; EBV: Epstein–Barr virus; eGFR: Estimated glomerular filtration rate; DBD: Donation after brain death; DCD: Donation after cardiac death; HLA: Human leukocyte antigen; IQR: Interquartile range; KRT: Kidney replacement therapy; LD: Living donor; SD: Standard deviation; uPCR: Urine protein–creatinine ratio.

## Data Availability

The data presented in this study are available upon request from the corresponding author (Rajkumar Chinnadurai).

## Supplementary Materials

The following supporting information can be downloaded at: https://www.mdpi.com/article/10.3390/jcm13071872/s1, Figure S1. Time from kidney transplantation to de novo post-transplant malignancy diagnosis; Table S1: Frequency distribution of primary kidney diseases listed in the ‘other’ category in relation to primary causes of end-stage kidney disease; Table S2: Organs and organ systems affected by de novo post-transplant malignancy; Table S3: Univariate Cox regression analysis evaluating risk factors for de novo post-transplant malignancy; Table S4: Cumulative incidence of death-censored graft loss amongst those who developed de novo post-transplant malignancy versus those who did not; Table S5: Cumulative incidence of transplant recipient death amongst those who developed de novo post-transplant malignancy versus those who did not; Table S6: Competing risk regression model of outcomes of death-censored graft loss with transplant recipient death as a competing risk; Table S7: Competing risk regression model of outcomes of transplant recipient death with graft loss as a competing risk.
