# Supplementary material for: Incidence, Risk Factors, and Outcomes of De Novo Malignancy following Kidney Transplantation
Source: J Clin Med. 2024 Mar 24;13(7):1872. doi: 10.3390/jcm13071872 (PMC11012944; doi:10.3390/jcm13071872)
Supplement: Supplementary file 1 [file jcm-13-01872-s001.zip › jcm-2895947-supplementary.pdf]

Supplementary Table and Figure File

Supplementary Table S1. Frequency distribution of primary kidney diseases listed in the 'other' category in relation to primary causes of end-stage kidney disease

Primary kidney diseases	Frequency
Acute kidney injury	5
Atheroembolic renal disease	2
Atrophic right kidney and hypertension	1
Atypical haemolytic uraemic syndrome	1
Bilateral nephrectomies	2
Bilateral kidney hypoplasia	1
Bilaterally small kidneys	2
Chronic kidney failure due to donor nephrectomy	1
CNI toxicity	1
Congenital dysplasia	7
Congenital neurogenic bladder	8
Congenital pelvic kidney	1
Congenital vesicoureteral junction obstruction	1
Cyclosporin	1
Cystinosis	2
Dent disease	1
Dysplastic kidneys	1
Fabry disease	1
Familial nephropathy	2
Horseshoe kidney	1
Hypercalcaemic nephropathy	2
Inherited aminoaciduria	1
Interstitial nephritis	1
Ischaemic renal disease	1
Lawrence-Moon-Biedl / Bardet-Biedl syndrome	1
Lithium induced nephropathy	3
Multifactorial CKD	1
Myeloma kidney	1
Nephropathy due to analgesic drugs	1
Nephropathy due to preeclampsia	2
Nephropathy related to HIV	1
Obstructive uropathy	3
Posterior urethral valves	4
Recurrent UTIs	1
Renal amyloidosis	1
Kidney hypoplasia	1
Single kidney identified in adulthood	5

Primary kidney diseases	Frequency
Small vessel disease	1
Syndrome of agenesis of abdominal muscles / Prune belly syndrome	1
Systemic vasculitis with ANCA negativity histologically proven	1
Traumatic injury to the kidney requiring emergency nephrectomy	1
Tubular interstitial nephritis	14
**Total**	**89**

ANCA: Antineutrophil cytoplasmic antibody; CKD: Chronic kidney disease; CNI: Calcineurin inhibitor; HIV: Human immunodeficiency virus; UTI: Urinary tract infection

Supplementary Table S2. Organs and organ systems affected by *de novo* post-transplant malignancy

De novo **post-transplant malignancy**	**Total (n = 963)**
**Organs involved**	**n (%)**
Lymphoid tissue	16 (20.5%)
Bowels	13 (16.7%)
Prostate	10 (12.8%)
Lung	9 (11.5%)
Kidney	9 (11.5%)
Breast	5 (6.4%)
Oesophageal	4 (5.1%)
Urothelial	3 (3.8%)
Vulva	2 (2.6%)
Tongue	2 (2.6%)
Liver	1 (1.3%)
Skin (Melanoma)	1 (1.3%)
Thyroid	1 (1.3%)
Pancreas	1 (1.3%)
Central nervous system	1 (1.3%)
**Organ systems involved**	**n (%)**
Genitourinary	24 (30.8%)
Gastrointestinal Tract	20 (25.6%)
Lymphoproliferative	16 (20.5%)
Respiratory	9 (11.5%)
Breast	5 (6.4%)
Endocrine	2 (2.6%)
Central nervous system	1 (1.3%)
Melanoma	1 (1.3%)

Supplementary Table S3. Univariate Cox regression analysis evaluating risk factors for *de novo* post-transplant malignancy

Characteristics	Hazard ratio	95%CI	p-value
Age (per decade)	1.59	1.34-1.88	**<0.001**
**Age (years) categories**			
< 30y	1	0.00-0.00	–
30-40y	1.25	0.44-3.51	0.675
40-50y	2.09	0.81-5.39	0.128
50-60y	4.09	1.66-10.04	**0.002**
60-70y	4.58	1.78-11.77	**0.002**
> 70y	9.34	3.19-27.39	**<0.001**
**Gender (Male)**	1.7	1.04-2.78	**0.036**
**Ethnicity**			
White	1	0.00-0.00	–
Black	0.66	0.09-4.73	0.677
Asian	0.66	0.30-1.43	0.290
Other	0.44	0.06-3.14	0.409
BMI	1	0.97-1.04	0.241
**Number of transplants**	0.94	0.52-1.72	0.849
**Pre-transplant diabetes**	1.3	0.73-2.32	0.375
**Total ischaemia time**	0.99	0.96-1.02	0.624
**Primary aetiology of kidney disease**			
ADPKD	1	0.00-0.00	–
GN	2.11	0.88-5.09	0.0948
DKD	1.93	0.67-5.58	0.2241
HKD	1.09	0.27-4.38	0.8984
Reflux/CPN	0.94	0.32-2.79	0.9092
Unknown	1.5	0.56-3.99	0.4217
Other (supplementary table 3)	2.34	0.88-6.23	0.0898
**Donor type**			
Deceased donor	1	0.00-0.00	–
Living donor	0.77	0.45-1.30	0.300
**Pre-emptive transplant**	0.81	0.49-1.34	0.400
**Total HLA mismatch**	1.09	0.92-1.29	0.300
**CMV donor (D) and recipient (R) status**			
CMV D+R+	1	0.00-0.00	–
CMV D+R-	1.24	0.65-2.36	0.520
CMV D-R-	0.6	0.27-1.30	0.193
CMV D-R+	0.58	0.26-1.31	0.192
**Duration on dialysis**	1	1.00-1.01	0.541
**Post-transplant diabetes**	0.67	0.35-1.31	0.245
**Antimetabolite**			
None	1	0.00-0.00	–

MPA	1.15	0.59-2.24	0.70
Azathioprine	1.45	067-3.14	0.30
**Corticosteroid treatment**			
Steroids given for < 2w	1	0.00-0.00	–
Steroid given for 2w-6mo	0.96	0.13-7.02	0.965
Steroid maintained > 6mo	1.32	0.83-2.09	0.241
**Donor CMV Positive**	1.51	0.89-2.56	0.123
**Recipient CMV Positive**	0.99	0.59-1.66	0.964
**History of Acute rejection**	1.45	0.67-3.16	0.300
**Smoking history**			
Never smoked	1	0.00-0.00	–
Ex-smoker	1.7	0.98-2.93	0.0582
Current smoker	1.06	0.52-2.16	0.8816
**Average CRP**	1.02	1.01-1.02	**<0.001**
**Baseline eGFR (ml/min/1.73m^2)**			
Baseline eGFR <30	1	0.00-0.00	–
Baseline eGFR 30-45	0.86	0.37-1.99	0.725
Baseline eGFR 45-60	0.69	0.30-1.60	0.391
Baseline eGFR 60-90	0.52	0.21-1.29	0.159
Baseline eGFR >90	0.29	0.06-1.39	0.121
**EBV infection**	2.09	1.28-3.41	**0.003**
**CMV infection**	1.4	0.77-2.53	0.273
**Polyoma virus infection**	0.72	0.31-1.66	0.445
**Average tacrolimus level**	1.03	0.95-1.11	0.452

ADPKD: Autosomal dominant polycystic kidney disease; BMI: Body mass index; CMV: Cytomegalovirus; CRP: C-reactive protein; CPN: Chronic pyelonephritis; DKD: Diabetic kidney disease; EBV: Epstein-Barr virus; eGFR: Estimated glomerular filtration rate; GN: Glomerulonephritis; HLA: Human leukocyte antigen; HKD: Hereditary kidney disease; MPA: Mycophenolic Acid; NODAT: New-onset diabetes after transplantation.

Supplementary Figure S1. Time from kidney transplantation to *de novo* post-transplant malignancy diagnosis

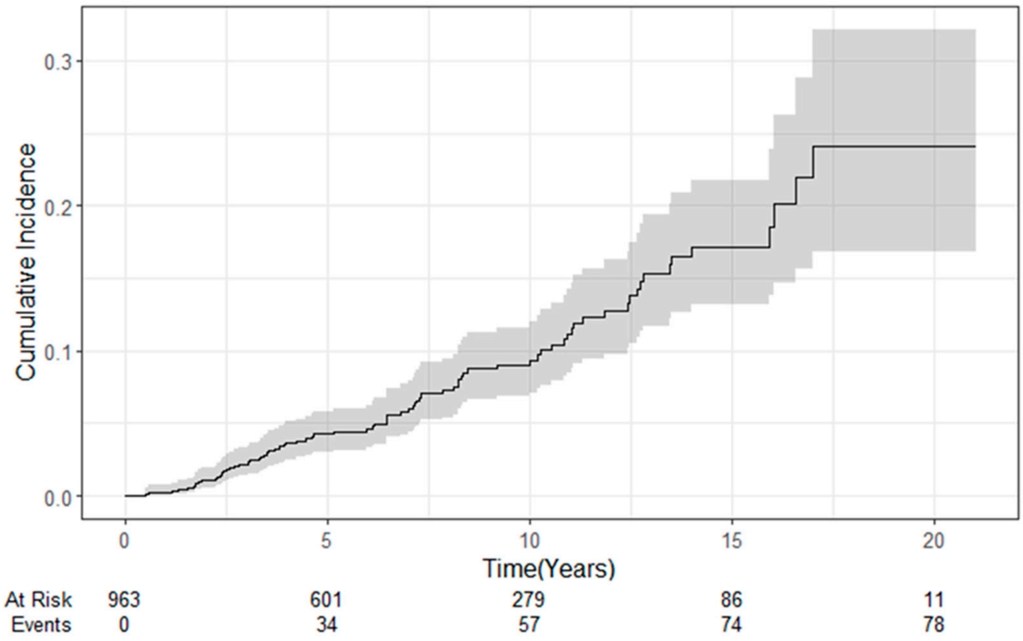

Supplementary Table S4. Cumulative incidence of death-censored graft loss amongst those who developed *de novo* post-transplant malignancy versus those who did not

Characteristic	*n*	5-year follow-up	10-year follow-up	15-year follow-up	p-value[1]
**DPTM**	**963**				0.5
No		3.4% (2.3% - 4.8%)	11% (8.3% - 14%)	17% (13% - 21%)	
Yes		2.6% (0.5% - 8.3%)	5.7% (1.8% - 13%)	16% (7.7% - 27%)	

[1]Gray's test

DPTM: *De novo* post-transplant malignancy

Supplementary Table S5. Cumulative incidence of transplant recipient death amongst those who developed *de novo* post-transplant malignancy versus those who did not

Characteristic	*n*	5-year follow-up	10-year follow-up	15-year follow-up	p-value[1]
**DPTM**	**963**				**0.002**
No		8.3% (6.5% - 10%)	20% (17% - 24%)	30% (25% - 35%)	
Yes		14% (7.6% - 23%)	32% (22% - 43%)	48% (34% - 60%)	

[1]Gray's test
DPTM: *De novo* post-transplant malignancy

Supplementary Table S6. Competing risk regression model of outcomes of death-censored graft loss with transplant recipient death as a competing risk

Co-variates	Age adjusted SHR (95%CI, p-value)	Age unadjusted SHR (95%CI, p-value)
Malignant neoplasia	1.31 (95%CI 0.70 - 2.49, p=0.400)	1.03 (95%CI 0.53 - 2.02, p=0.920)
Recipient age (per decade)	**0.67 (95%CI 0.56 - 0.80, p<0.001)**	_
Gender (Male)	0.94 (95%CI 0.53 - 1.64, p=0.820)	1.00 (95%CI 0.59 - 1.71, p=0.990)
**Primary aetiology of kidney disease**		
ADPKD	Reference	Reference
Unknown	1.20 (95%CI 0.38 - 3.74, p=0.760)	1.47 (95%CI 0.47 - 4.63, p=0.510)
GN	2.06 (95%CI 0.78 - 5.43, p=0.140)	2.52 (95%CI 0.98 - 6.52, p=0.056)
Reflux/CPN	1.65 (95%CI 0.53 - 5.15, p=0.390)	2.07 (95%CI 0.70 - 6.17, p=0.190)
DKD	2.37 (95%CI 0.70 - 7.93, p=0.160)	2.20 (95%CI 0.69 - 7.06, p=0.180)
Other	1.39 (95%CI 0.41 - 4.66, p=0.600)	1.83 (95%CI 0.56 - 6.00, p=0.320)
HKD	2.13 (95%CI 0.51 - 8.99, p=0.300)	1.93 (95%CI 0.50 - 7.41, p=0.340)
Deceased donor	1.25 (95%CI 0.74 - 2.12, p=0.410)	0.90 (95%CI 0.53 - 1.53, p=0.710)
Total HLA mismatch	1.10 (95%CI 0.90 - 1.35, p=0.370)	1.04 (95%CI 0.86 - 1.24, p=0.720)
History of Acute rejection	**3.53 (95%CI 2.06 - 6.03, p<0.001)**	**3.95 (95%CI 2.34 - 6.65, p<0.001)**
Baseline eGFR (per 10ml/min/1.73m^2 increase)	0.85 (95%CI 0.71 - 1.01, p=0.064)	0.94 (95%CI 0.81 - 1.08, p=0.350)

ADPKD: Autosomal dominant polycystic kidney disease; eGFR: Estimated glomerular filtration rate; GN: glomerulonephritis; CPN: chronic pyelonephritis; DKD: Diabetic kidney disease; HKD: Hypertensive kidney disease; HLA: Human leucocyte antigen; SHR: Sub-hazard ratio

Supplementary Table S7. Competing risk regression model of outcomes of transplant recipient death with graft loss as a competing risk

Co-variates	Age adjusted SHR (95%CI, p-value)	Age unadjusted SHR (95%CI, p-value)
Malignant neoplasia	1.31 (95%CI 0.86 - 1.99, p=0.210)	**1.78 (95%CI 1.19 - 2.66, p=0.005)**
Recipient Age (per decade)	**1.77 (95%CI 1.54 - 2.03, p<0.001)**	–
Gender (Male)	1.34 (95%CI 0.93 - 1.92, p=0.11)	1.23 (95%CI 0.86 - 1.77, p=0.260)
**Primary aetiology of kidney disease**		
ADPKD	Reference	Reference
Unknown	1.47 (95%CI 0.78 - 2.77, p=0.240)	1.12 (95%CI 0.59 - 2.13, p=0.720)
GN	1.34 (95%CI 0.75 - 2.40, p=0.320)	1.08 (95%CI 0.60 - 1.94, p=0.800)
Reflux/CPN	1.10 (95%CI 0.59- 2.06, p=0.770)	1.01 (95%CI 0.53 - 1.92, p=0.970)
DKD	**2.57 (95%CI 1.36 - 4.83, p=0.004)**	**2.77 (95%CI 1.45 - 5.30, p=0.002)**
Other	1.49 (95%CI 0.72 - 3.10, p=0.290)	1.15 (95%CI 0.57 - 2.35, p=0.700)
HKD	1.52 (95%CI 0.73- 3.19, p=0.270)	1.82 (95%CI 0.87 - 3.81, p=0.110)
Pre-emptive transplant	**0.56 (95%CI 0.39 - 0.82, p=0.002)**	**0.55 (95%CI 0.37 - 0.80, p=0.002)**
Baseline eGFR (per 10ml/min/1.73m^2 increase)	0.94 (95%CI 0.86 - 1.02, p=0.150)	**0.87 (95%CI 0.79 - 0.96, p=0.005)**

ADPKD: Autosomal dominant polycystic kidney disease; eGFR: Estimated glomerular filtration rate; GN: glomerulonephritis; CPN: chronic pyelonephritis; DKD: Diabetic kidney disease; HKD: Hypertensive kidney disease; HLA: Human leucocyte antigen; SHR: Sub-hazard ratio
